# Tracking and Controlling the Spatiotemporal Spread of SARS‐CoV‐2 Lineage B.1.1.7 in COVID‐19 Reopenings

**DOI:** 10.1029/2021GH000517

**Published:** 2021-12-11

**Authors:** Chengzhuo Tong, Wenzhong Shi, Anshu Zhang, Zhicheng Shi

**Affiliations:** ^1^ Department of Land Surveying and Geo‐Informatics Otto Poon Charitable Foundation Smart Cities Research Institute The Hong Kong Polytechnic University Hong Kong China; ^2^ Research Institute for Smart Cities School of Architecture and Urban Planning Shenzhen University Shenzhen China

## Abstract

Understanding why or how the emergence of SARS‐CoV‐2 variants has occurred and how to control them is crucial as regards the potential of global reopening. To explore and further understand the spatiotemporal dynamics of the B.1.1.7 spread in the 368 districts of Taiwan, a district‐level geographic prediction model of the risk of COVID‐19 symptom onset has been proposed. It has been found that, (a) the human mobility, epidemic alert measures, and vaccination rates all played an important role in the spatiotemporal heterogeneity of B.1.1.7 transmission; (b) for regions with high human mobility and low vaccination rates, the partial relaxation of entry quarantine measures for specific imported groups would, in fact, lead to a wide spread of B.1.1.7 with a consequent doubling of high‐onset‐risk areas and together with the overall onset risk, a further increase of more than 20% would occur; (c) compared with the closing of business places and public venues in all districts, both lockdown in those areas of high‐onset‐risk and the gathered control effects regarding other districts, the control of B.1.1.7 spread would be better enabled by an onset risk reduction of up to 91.36%. Additionally, an increase in the vaccination rate in each district by up to 5–10 times would further reduce the onset risk by 6.07%–62.22%.

## Key Points

- The partial relaxation of entry quarantine led to a wide spread of B.1.1.7 in regions with high human mobility and low vaccination rates
- Both lockdown in high‐onset‐risk districts and the gathered control in other districts better enabled the control of the B.1.1.7 spread
- When the vaccination rates were raised to a certain higher level, the spatiotemporal spread of B.1.1.7 could be controlled more effectively

## 1 Introduction

The SARS‐CoV‐2 lineage B.1.1.7 (Alpha) has spread to 114 countries around the world since it was first detected in the UK in September 2020 (P. Wang, Nair, et al., 2021). Some studies have shown that B.1.1.7 is not only 43%–90% (95% CI 38–130) more transmissible than preexisting variants (Jewell, 2021; Walensky et al., 2021) but also causes more severe illness (Davies et al., 2021; Patone et al., 2021). With the accelerated vaccination, the world is gradually entering a regular prevention and control stage (Pham et al., 2021) with some regions preparing to reopen to the world (Chang et al., 2021; Zhao et al., 2021). In order to assist these regions to effectively prevent the emergence of new variants in the reopening process, it became crucial to explore the nonpharmaceutical intervention (NPI) measures that affect the emergence and spread of SARS‐CoV‐2 variants such as B.1.1.7 and thereby, first learn in general how to effectively control the spatiotemporal spread of SARS‐CoV‐2 variants. Taiwan, one of the regions where B.1.1.7 appeared during the reopening process (Shonchoy et al., 2021; Tan, 2021; Yu et al., 2021), is chosen regarding the exploration of the spatial dynamics of the B.1.1.7 spread and investigation with the aim of exploring relevant data and thereby finding the potential means to effectively control the spatiotemporal spread of B.1.1.7, potentially by the integration of vaccination and NPI measures.

From early 2020 to the end of March 2021, Taiwan had less than 77 local COVID‐19 cases and only 10 deaths (Taiwan Centers for Disease Control, 2021c). By shutting its borders early and requiring 2‐week quarantines of almost everyone arriving from overseas (Chiu et al., 2021; Lin et al., 2020; Ng et al., 2021), Taiwan managed to keep life on the island mostly unfettered (Chien et al., 2020; Dai et al., 2021; Hwang et al., 2020). However, that success came to an end in early April 2021 after cases appeared that carried the COVID‐19‐variant B.1.1.7 (Tan, 2021), resulting in an increase of more than 7,045 local onset cases in 62 days as of 5 June 2021 (Taiwan Centers for Disease Control, 2021b). Taiwan's COVID‐19 case fatality rate subsequently reached 4.9%, the eighth highest worldwide (South China Morning Post, 2021), after the emergence of B.1.1.7. This was more than twice the global average COVID‐19 fatality rate (Worldometer, 2021). The latest gene sequencing results (Central News Agency, 2021) show that B.1.1.7 has become the dominant variant from the south to the north of Taiwan. Prior to the emergence of the B.1.1.7, Taiwan, in early April 2021 (Taiwan Centers for Disease Control, 2021f), had begun to gradually relax the previously enforced strict prevention measures for specific groups and the region continued to open up. Oxford/AstraZeneca vaccinations were organized for specific groups in 22 cities (Taiwan Centers for Disease Control, 2021d). However, after the emergence of the B.1.1.7, Taiwan raised the epidemic alert level to Level 2 (Focus Taiwan, 2021) (e.g., social distancing for public gathering and crowd control) and Level 3 (Focus Taiwan, 2021) (e.g., closing all places of business and public venues).

At this point in time (beginning of May), the district‐level weighted kernel density estimation (WKDE) model was proposed to predict the spatiotemporal COVID‐19 symptom onset risk (Shi, Tong, Zhang, & Shi, 2021; Shi, Tong, Zhang, Wang, et al., 2021) and to further explore the spread of B.1.1.7 in 368 districts (i.e., township, county‐administered city, and district) of Taiwan. The daily human mobility (Apple, 2021; Google, 2021), vaccination rates (The Center for High‐performance Computing, 2021), and social distancing‐level factors (Badr et al., 2020; Cot et al., 2021; Woskie et al., 2021) in 368 districts were incorporated to enhance the model. Thus, based on the above onset risk prediction results, the impacts of relaxation and tightened prevention measures regarding the emergence and the spread of B.1.1.7 are to be investigated in this study. The spatiotemporal spread of B.1.17 at different epidemic alert levels and vaccination levels is to be simulated. The spatiotemporal data of the daily onset cases in 368 districts of Taiwan from 5 April 2021 to 5 June 2021 are utilized in the development of this study.

## 2 Materials and Methods

### 2.1 Data Sources

**Figure 1:**
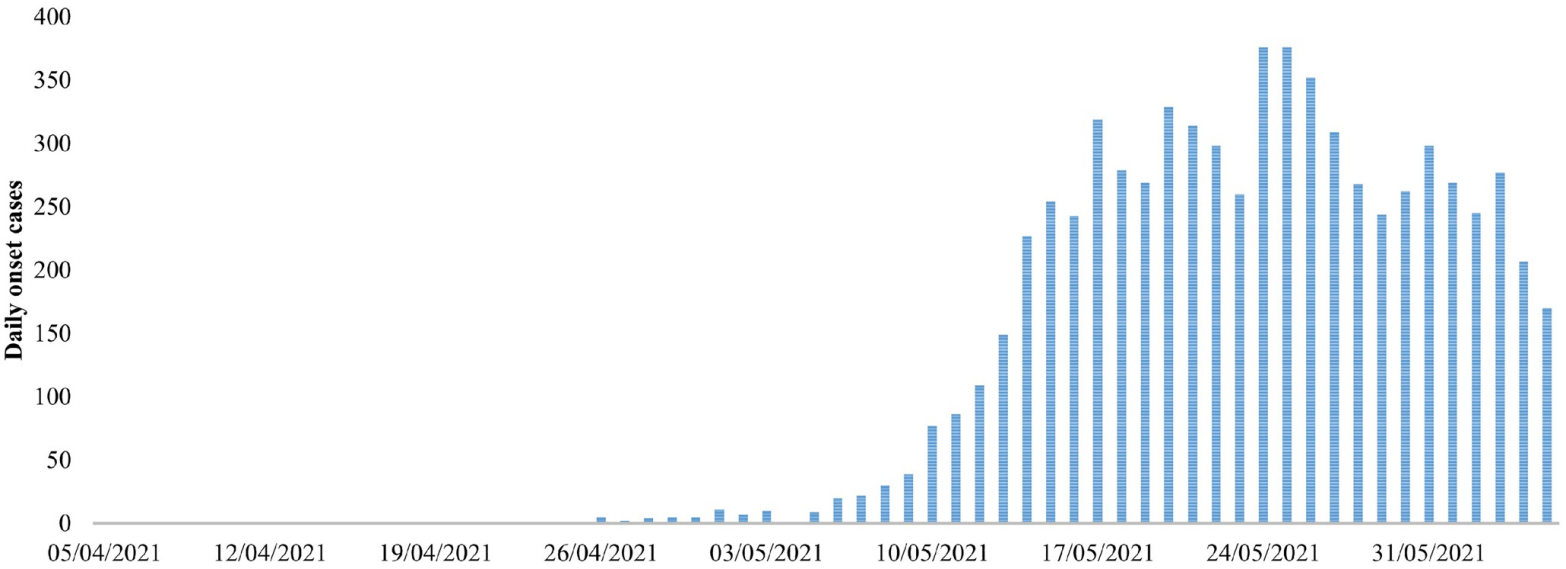
The daily variation in COVID‐19 symptom onset cases in Taiwan from 5 April to 5 June 2021.

A total of 7,045 COVID‐19 onset cases with spatiotemporal information in Taiwan during the period from 5 April to 5 June 2021 were collected from official reports (Taiwan Centers for Disease Control, 2021a) by Centers for Disease Control (Figure 1). For these 7,045 cases, the available information on the dates of onset was obtained together with the district‐level locations in which these cases had been located prior to diagnosis.

**Figure 2:**
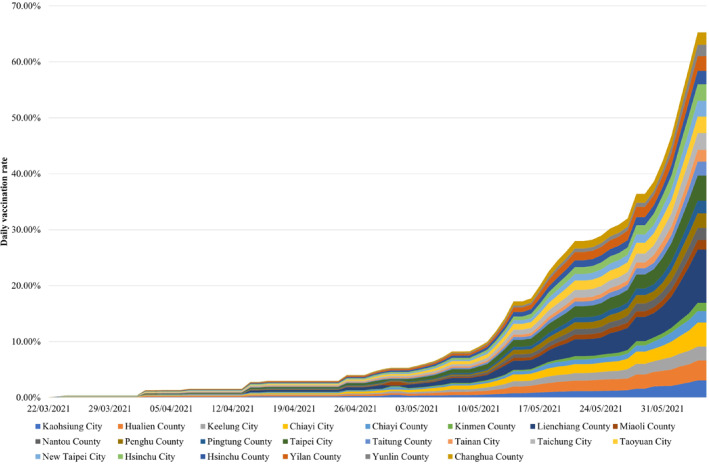
The daily variation rates of the Oxford/AstraZeneca vaccine in the 22 cities of Taiwan from 5 April to 5 June 2021.

In addition, to quantify the daily human mobility and the impact of the social distancing control level related to the COVID‐19 epidemic in all 22 cities of Taiwan, the daily human mobility trend data from 5 April to 5 June 2021 provided by Apple Map (Apple, 2021) and Google Map (Google, 2021) were used. Until 5 June 2021, the COVID‐19 vaccines used in Taiwan were all Oxford/AstraZeneca vaccines (The Center for High‐performance Computing, 2021). The clinical vaccine efficacy against symptomatic positive infection was 70.4% (95% CI 43.6–84.5) for B.1.1.7 (Emary et al., 2021). In order to measure the impact of vaccination on the COVID‐19 epidemic, daily vaccination rates (The Center for High‐performance Computing, 2021) from 5 April to 5 June 2021 were used (Figure 2). The data were collected from all the 22 cities of Taiwan.

### 2.2 A District‐Level WKDE Model for Predicting the Onset Risk of COVID‐19 Symptoms

It has been found that SARS‐CoV‐2 has a high viral load and transmissibility, following the date of symptom onset (Walsh et al., 2020). Thus, it is necessary to adopt data‐driven spatiotemporal models to dynamically and individually assess the degree of onset risk level. Shi et al. developed an intercity‐level extended WKDE model (Shi, Tong, Zhang, & Shi, 2021; Shi, Tong, Zhang, Wang, et al., 2021). The model performs a retrospective analysis based on the spatiotemporal information of onset cases to infer the infection date of each onset case, further infer the spatial distribution (that is, the kernel density surface) of the infected risk of people by the onset cases at a past date, and hence, finally predict the distribution of onset risk at a future date. In addition, according to the transmission law of COVID‐19 (i.e., mainly through direct, indirect, or close contact between people), the dynamic flow of people data was introduced into the model to improve the accuracy of prediction. This data‐driven spatial prediction method can reduce the dependence on theoretical assumptions and environmental parameters, which is suitable for the current stage, that is, when the transmission characteristics of SARS‐CoV‐2 variants are not very clear.

As a further development of the original extended WKDE model (Shi, Tong, Zhang, & Shi, 2021; Shi, Tong, Zhang, Wang, et al., 2021), the district‐level WKDE model, proposed in this study, included the following three steps:

1) Conducting a retrospective analysis of the historical existence likelihood of the infection in each district location in which an onset case had stayed;
2) Making inferences on the historical existence likelihood of the infection in the entire region; and
3) Making predictions about the onset risk in the entire region on a given day in the near future.

The main difference between the district‐level WKDE model and the original extended WKDE model is that at step 2) of the model, the historical existence likelihood of an infection in a random location in the entire region was formulated as follows:

(1) $$P_{\text{Infection}}\left(S,t_{i}\right)=n{\left(t_{i}\right)}^{-1}\sum_{j=1}^{n\left(ti\right)}\frac{1}{V\left(S,t_{i}\right)}M_{SD}\left(S,t_{i}\right)M\left(S,t_{i}\right)P_{\text{Infection}}\left(L_{j},\;t_{i}\right)K_{h}\left(S-L_{j}\right)$$

where *P*_Infection_(*S*, *t*_*i*_) is the probability of any individual infected with COVID‐19 and hence infecting others in a random location, *S*, in the city on day *t*_*i*_. All the days in this study period (5 April to 5 June 2021) are in the order denoted as *t*_1_, *t*_2_,… *t*_65_. The range of *i* is 1–65; *n* is the total number of onset cases in all 65 days. *n*(*t*_i_) denotes the number of onset cases on day *t*_i_; *L*_*j*_ is the *j*th location among the district places where the onset cases resided; *P*_Infection_(*L*_*j*_, *t*_*i*_) denotes the probability that one onset case was infected on day *t*_*i*_ in the location *L*_*j*_ *;* and *K*_*h*_(*S*–*L*_*j*_) denotes a Gaussian kernel between locations *S* and *L*_*j*_. The values of *P*_Infection_ (*L*_*j*_, *t*_*i*_), *K*_*h*_ (*S*–*L*_*j*_), and *h* were determined in the earlier model procedures (Shi, Tong, Zhang, & Shi, 2021; Shi, Tong, Zhang, Wang, et al., 2021).

*V*(*S*, *t*_*i*_) denotes the cumulative vaccination rate in the city containing the location *S* on day *t*_*i*_.

(2) $$V\left(S,t_{i}\right)=i^{-1}V_{k}$$

where *V*_*k*_ denotes the daily vaccination rate of the city containing location *S* on day *t*_*k*_ prior to *t*_i_.

*M*(*S, t*_*i*_) denotes the human mobility factor at location *S* on day *t*_*i*_ calculated as

(3) $$M\left(S,t_{i}\right)=i^{-1}M_{k}$$

where *M*_*k*_ denotes the daily human mobility of the city containing location *S* on day *t*_*k*_ prior to *t*_*i*_.

*M*_*SD*_(*S*, *t*_*i*_) denotes the social distancing‐level factor in the city on day *t*_*i*_ calculated as follows (Badr et al., 2020):

(4) $$M_{SD}\left(S,\text{ti}\right)=i^{-1}\sum_{k=1}^{i}\frac{M_{k}}{M_{t0}}$$

where *M*_*k*_ denotes the daily human mobility in a city containing the location *S* on day *t*_*k*_ prior to *t*_*i*_; *M*_*t0*_ is the average value of the daily human mobility data in the specific city before the COVID‐19 outbreak date; and $M_{k}/M_{t0}$ denotes the amount of daily social distancing in a city containing location *S* on day *t*_*k*_ prior to *t*_*i*._

In particular, for the new scenario of the Level 4 epidemic alert for Taipei and New Taipei City and the Level 2 epidemic alert for the remaining 19 cities, the daily human mobility M(*S*, *t*_*i*_) and social distancing‐level factor M_*SD*_(*S, t*_*i*_) for districts in Taipei and New Taipei were set as 0 after 15 May 2021. The daily human mobility and social distancing‐level factors for districts in remaining 19 cities were set as usual before 15 May 2021. For the new scenario of the Level 2 epidemic alert maintained after 15 May 2021, the daily human mobility M(*S*, *t*_*i*_) and social distancing‐level factor M_*SD*_(*S, t*_*i*_) for all 368 districts were set as usual before 15 May 2021.

Finally, the predicted risk of COVID‐19 symptom onset in each location (i.e., the likelihood that at least one person infected by an onset case at each location develops clinical symptoms on a future day) was divided by the maximum predicted risk among all locations on a specific date and thereby standardized to a value between 0 and 1 (Shi, Tong, Zhang, & Shi, 2021; Shi, Tong, Zhang, Wang, et al., 2021). Different levels of onset risk were established as follows: low onset risk [0–0.2], low‐medium onset risk [0.2–0.4], medium onset risk [0.4–0.6], medium‐high onset risk [0.6–0.8], and high onset risk [0.8–1]. The accuracy of the predicted risk of symptom onset was evaluated daily by calculating the percentage of onset cases reported in areas in which the predicted symptom onset risk was >0.8 (identified as onset hot spots; Shi, Tong, Zhang, & Shi, 2021; Shi, Tong, Zhang, Wang, et al., 2021). For example, if 100 cases occurred in the entire study area on the date for which the prediction is made and out of which 70 cases occurring in the areas with predicted onset risk over 0.8, then the prediction accuracy on that date was 70%. This definition of the prediction accuracy followed the idea of hit rate (Bowers et al., 2004; Hart & Zandbergen, 2013), the popular indicator for the prediction accuracy of the Kernel Density Estimation model on which the district‐level WKDE model was based.

### 2.3 Geodetector

The Geodetector method is usually used to measure the degree of spatial stratified heterogeneity and test its significance through the within‐strata variance less than the between‐strata variance. To explore the degree of the determinant power of the selected risk factor (mobility, epidemic alert levels, and vaccination rates) in the spatial stratified heterogeneity of the B.1.17 spread (J. F. Wang et al., 2016; Xu et al., 2021), the factor detection in the GeoDetector is conducted by q‐statistic (J. F. Wang et al., 2010; L. Wang, Xu, et al., 2021):

(5) $$\begin{array}{l} q=1-\frac{\sum_{h=1}^{L}N_{h}\sigma_{h}^{2}}{N_{\sigma^{2}}}\\ \sigma^{2}=\frac{1}{N}{\sum_{i=1}^{N}\left(Ri-\bar{R}\right)}^{2}\\ {\sigma_{h}}^{2}=\frac{1}{N}{\sum_{j=1}^{N_{h}}\left(R_{h,j}-\bar{R_{h}}\right)}^{2} \end{array}$$

where *q* is the explanatory power of factor *X* on the spatial heterogeneity of the factor *Y* (i.e., the predicted risk of COVID‐19 symptom onset). *σ* ^2^ and *σ*_*h*_^2^ represent the variance of the spatial risk of the COVID‐19 symptom onset in the whole region in *N* districts and in the *h*th stratum in *N*_*h*_ districts, respectively (L. Wang, Xu, et al., 2021). The parameters *R*_*i*_ and *R*_*h,j*_ represent the spatial risk of the COVID‐19 symptom onset in the *i*th district and the *j*th district in the *h*th stratum, respectively (L. Wang, Xu, et al., 2021). $\bar{R}$ and $\bar{R_{h}}$ refer to the respective average risk of the COVID‐19 symptom onset within the whole study region and a specific stratum (L. Wang, Xu, et al., 2021). The value of *q* ranges from 0 to 1 (J. F. Wang et al., 2010). The larger the value of *q*, the stronger the effect of the factor *X* on the factor *Y* (J. F. Wang et al., 2010).

## 3 Results

### 3.1 A District‐Level WKDE Model for Predicting the Onset Risk of COVID‐19 Symptoms

**Figure 3:**
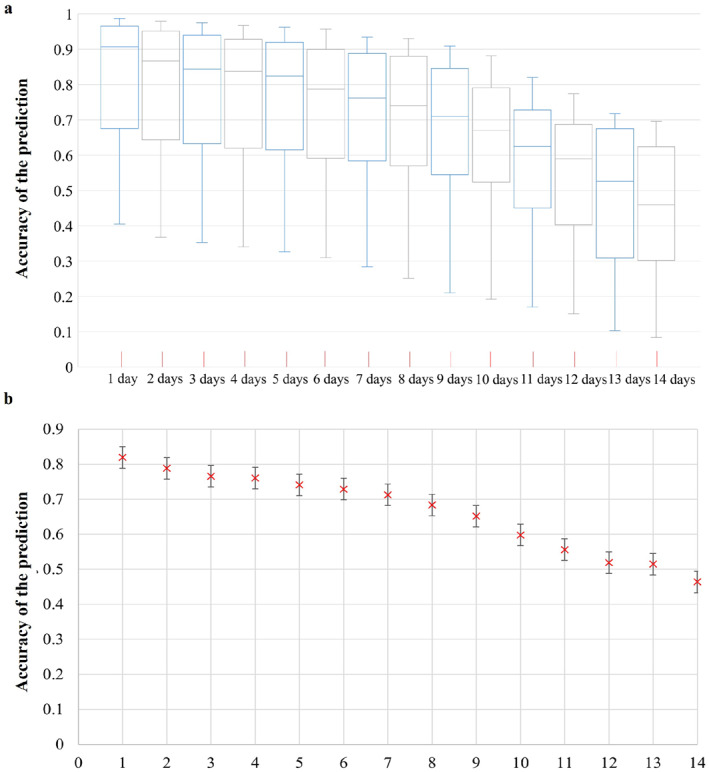
The accuracy of the predicted risk of COVID‐19 symptom onset by district‐level weighted kernel density estimation (WKDE) models and 95% confidence interval of the prediction accuracy. (a) Accuracy of the predicted risk of COVID‐19 symptom onset by district‐level WKDE models. The predicted onset risk is a normalized value between 0 and 1, hence indicating the risk relative to the highest predicted risk among all locations on the date for which the risk of symptom onset is predicted, hereafter termed “the prediction date.” The prediction accuracy is defined, on the prediction date, as the percentage of the onset cases in the areas in which the predicted onset risk was higher than 0.8 (identified as onset hot spots). The time interval denotes the period between the base date and the date of prediction. The horizontal line in the box denotes the median, while the lower and upper edges of the box represent the first and third quartiles, respectively. The lines emanating from the box upward and downward represent the maximum and minimum values, respectively. (b) 95% confidence interval of the mean accuracy of the predicted risk of COVID‐19 symptom onset by district‐level WKDE models.

The COVID‐19 symptom onset risk in 368 Taiwan districts, during the whole process of SARS‐CoV‐2 lineage B.1.1.7 emergence and spread, was first predicted and analyzed using the district‐level WKDE model. A total of 7,045 COVID‐19 onset cases with spatiotemporal information in Taiwan during the period from 5 April to 5 June 2021 were used in the model. In this study, the accuracy of the predicted risk of symptom onset was evaluated daily. The prediction accuracy was defined as the percentage of onset cases reported in hot spot areas in which the predicted risk of symptom onset was >0.8 (Table 1). The prediction accuracy of the district‐level WKDE model was over 75% regarding the prediction of the symptom onset risk during the following 7 days (Figure 3). Such an outperformance should be attributed to the incorporation of daily human mobility, vaccination rates, and social distancing‐level factors at the within‐city scale.

**Table 1:** The Relationship of Predicted Risk of COVID‐19 Symptom Onset Resulted From the District‐Level WKDE Model and the Number of Onset Cases in Different Districts

Predicted onset risk value in districts	Average number of onset cases per district
0–0.2	0.093
0.2–0.4	0.29
0.4–0.6	0.69
0.6–0.8	1.27
0.8–1	13.53

The spatiotemporal variations of the predicted onset risk are described as follows. On 6 April 2021, seven districts in the central part of the Greater Taipei (Taipei, New Taipei, Taoyuan, and Keelung), first began to be at the high onset risk (Figure 4a). These districts are also the initial primary source of B.1.1.7 (Central News Agency, 2021). The B.1.1.7 lineage further spread from the Greater Taipei, the center of the mobility network, to other important nodes in the mobility network of Taiwan (Figures 4b–4g). Thus, all seven main metropolitan areas (Directorate General of Budget, Accounting and Statistics, 1993), accounting for more than 74% of Taiwan's population (Department of Household Registration, 2021), were all at high onset risk. As a result, the overall onset risk in Taiwan reached the highest level (Figure 4h). In order to control the trend of further spread, Level 2 epidemic alert and Level 3 epidemic alert were implemented one after the other. However, after implementing the above measures, human mobility in other major cities remained at approximately 60%–98% of their usual level except for Taipei. Thus, more than 51% of the districts in the seven main metropolitan areas (Figure 4i) still remained at the high onset risk level for 2 weeks after implementing the Level 3 epidemic alert. However and of interest, the spatial spread of B.1.1.7 seems to have limited the impact on rural areas distant from the main mobility networks. The vast majority of rural areas in eastern Taiwan was at low‐medium risk during the entire epidemic.

Figure 4Predicted risk of COVID‐19 symptom onset across 368 Taiwan districts. (a–i) The predicted onset risk during the study period from 5 April to 5 June 2021. The predicted COVID‐19 symptoms onset risk was generated using the district‐level weighted kernel density estimation model.

**Figure:**
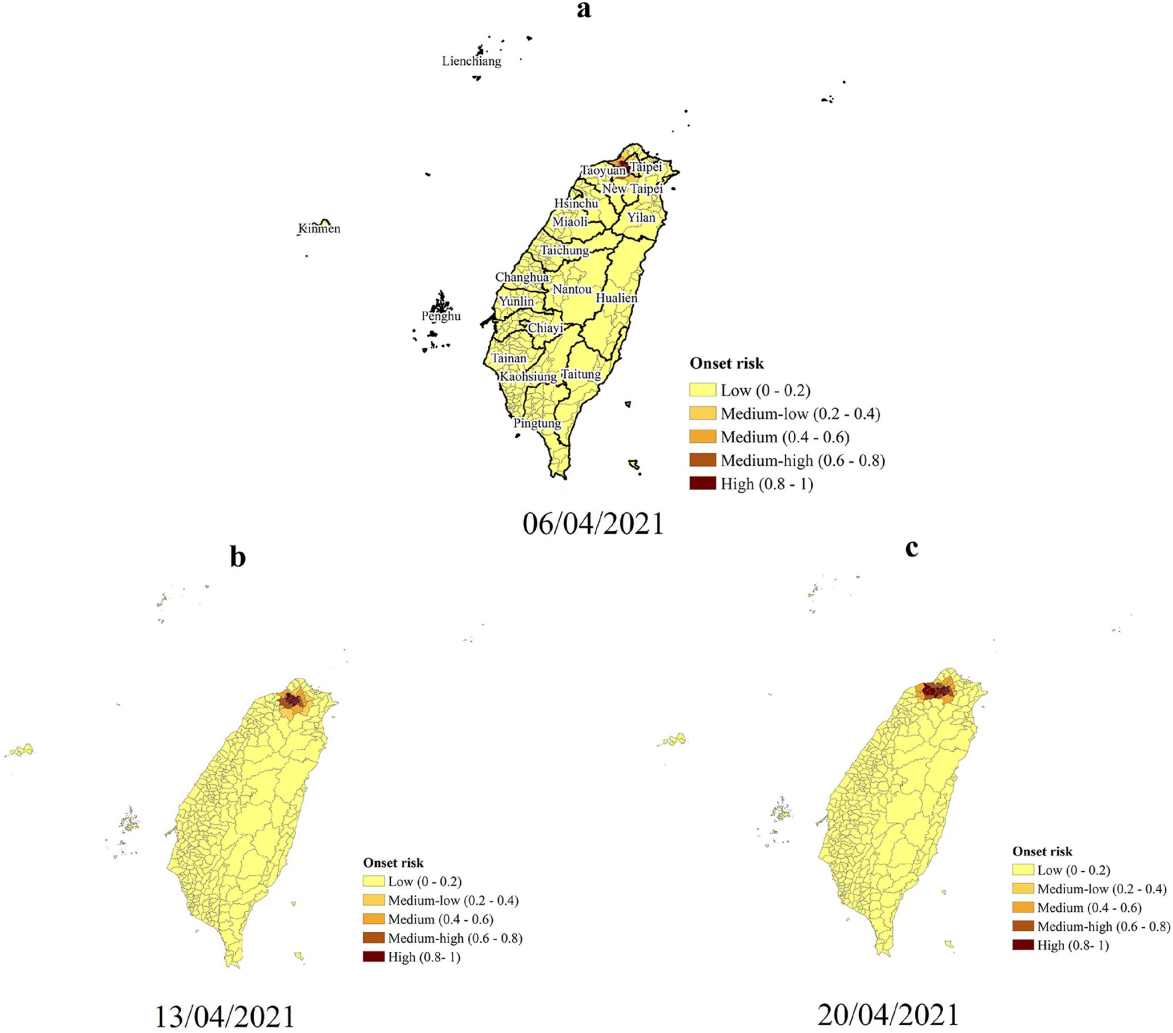

**Figure:**
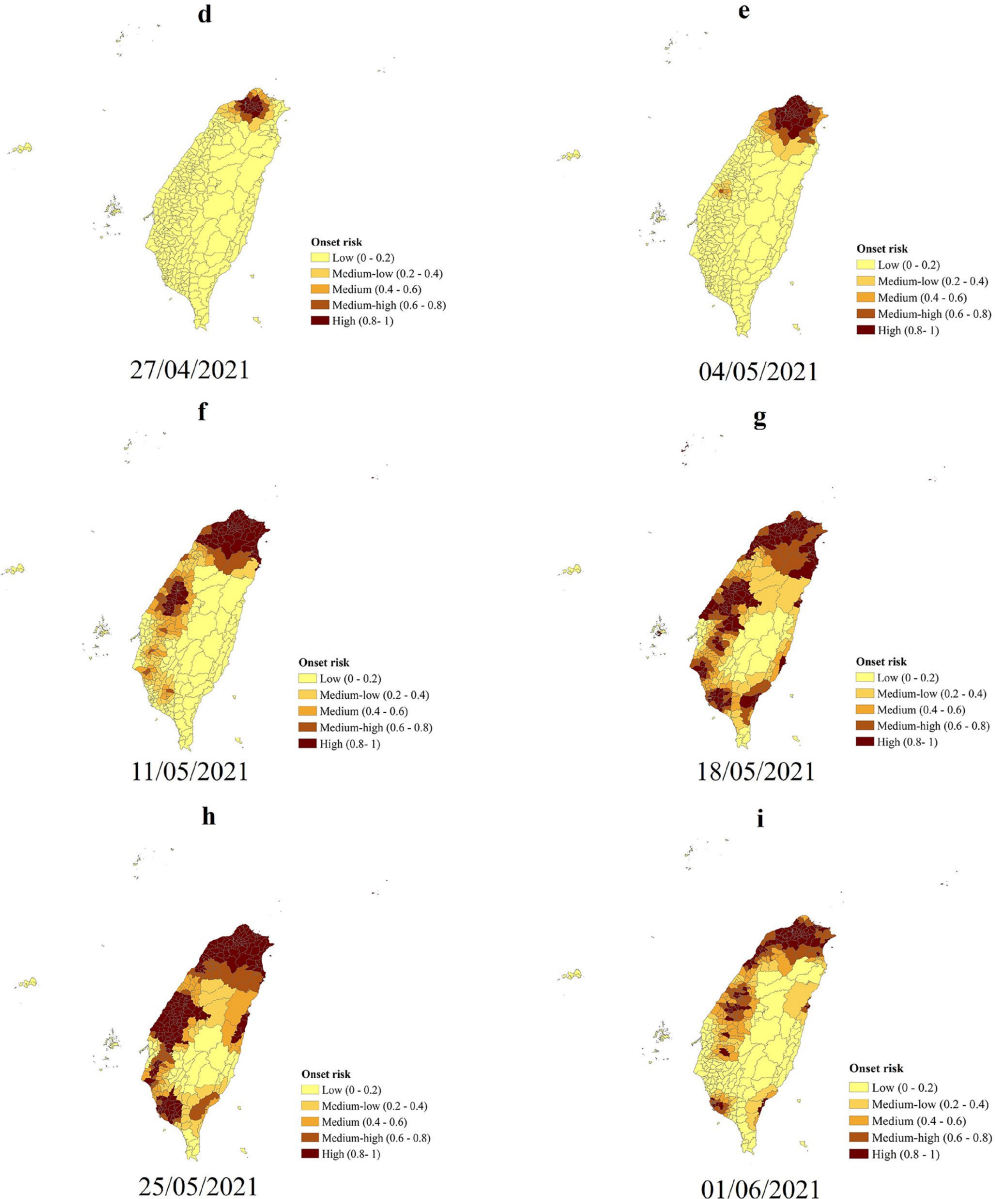

### 3.2 To Investigate the Impacts of Relaxation and Tightening Control Measures on the Spatiotemporal Spread of B.1.1.7

**Figure 5:**
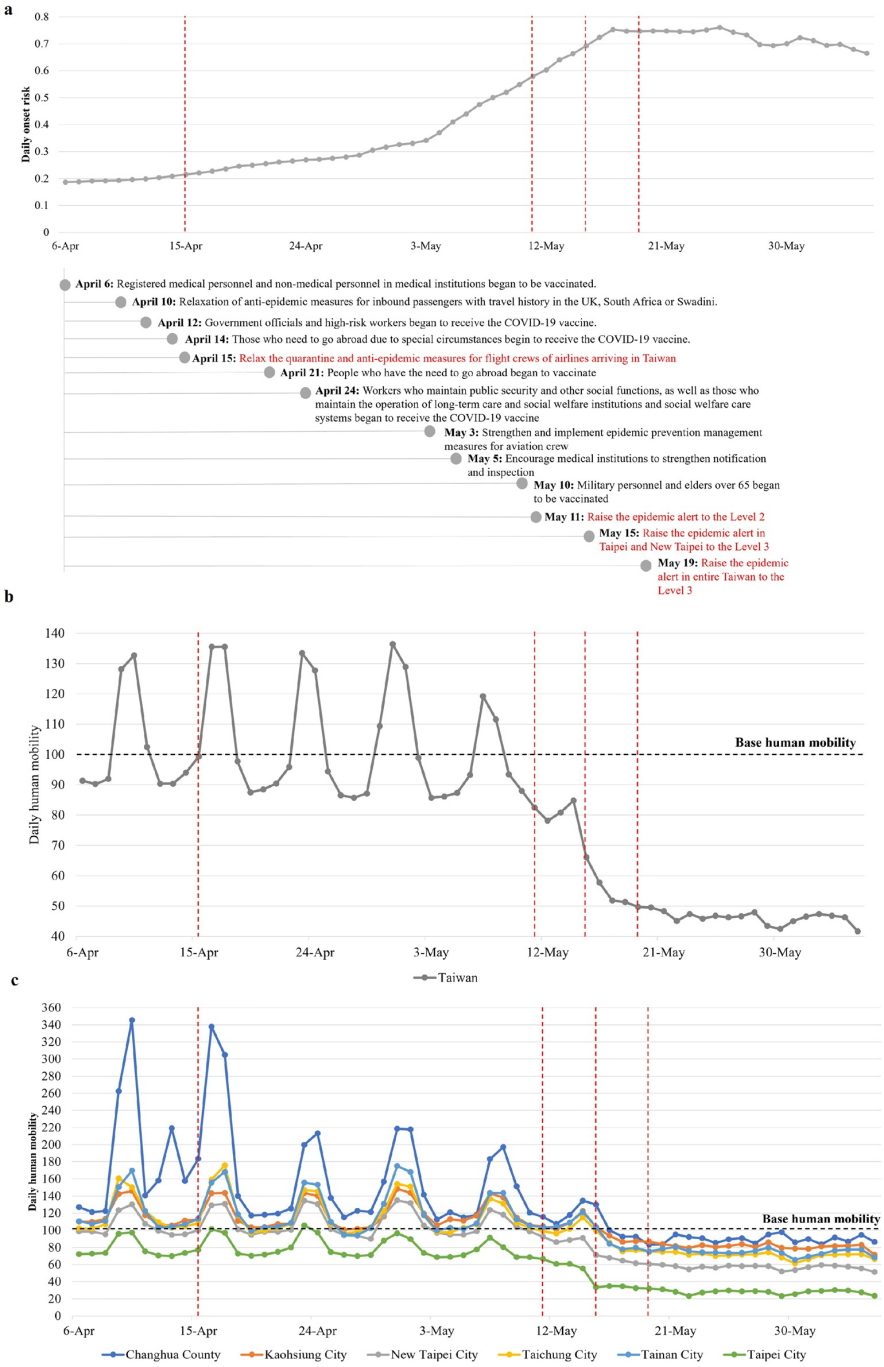
The overall risk of COVID‐19 symptom onset and the daily human mobility from 6 April to 5 June 2021. (a) The overall risk of COVID‐19 symptom onset in Taiwan from 6 April to 5 June 2021. The plotted values were computed from the predicted risk of COVID‐19 symptom onset in 368 districts in Taiwan. The four vertical red dashed lines from left to right indicate the measures of relaxing the quarantine and anti‐epidemic measures for airline flight crews (15 April 2021), raising the epidemic alert to the Level 2 (11 May 2021), raising the epidemic alert in Taipei and New Taipei to the Level 3 (15 May 2021), and raising the epidemic alert in entire Taiwan to the Level 3 (19 May 2021). (b) The daily overall human mobility in Taiwan from 6 April to 6 June 2021. The plotted values are the daily overall human mobility values in Taiwan. (c) The daily human mobility in six representative main cities of Taiwan from 6 April to 5 June 2021. The plotted values are the daily human mobility values in six main cities of Taiwan.

To further analyze the effects of relaxation and tightening the prevention measures regarding the emergence and spread of the B.1.1.7, daily overall onset risk after the implementation of the 13 primary measures in the whole process of B.1.1.7 spread was compared. The relaxation of measures taken began in early April, which raised Taiwan's overall onset risk level to a certain extent, especially the relaxation of the quarantine and anti‐epidemic measures for flight crews of those airlines arriving in Taiwan from 15 April 2021. After the implementation of this relaxation measure, the overall onset risk in the 368 districts of Taiwan increased by 21.40%, 41.86%, and 104.65% during those weeks: first (15–22 April 2021), second (22–29 April 2021), and final (29 April to 6 May 2021) (Figure 5a, Table 2). The area within the high onset risk level had also expanded by 1.07 times (Figure 5a). More seriously, before and during the process of easing the epidemic prevention measures, daily human mobility in Taiwan had constantly remained at more than 85% of the base human mobility (i.e., the usual human mobility before the epidemic) and sometimes even reaching 1.4 times the base human mobility (Figure 5b). Specifically, daily human mobility in some cities even reached 3.46 times the base human mobility (Figure 5c).

**Table 2:** The Increased Percentage of Overall Onset Risk in 368 Districts, From 15 April to 6 May 2021 After the Relaxation of the Quarantine and Anti‐Epidemic Measures for Flight Crews of Those Airlines Arriving in Taiwan

	15 April 2021	22 April 2021	29 April 2021	6 May 2021
The relaxation of the quarantine and anti‐epidemic measures for flight crews of those airlines arriving in Taiwan	0.215	0.261 (increase in overall onset risk: 21.40%)	0.305 (increase in overall onset risk: 41.86%)	0.44 (increase in overall onset risk: 104.65%)

Meanwhile, although Taiwan was also constantly opening up the Oxford/AstraZeneca vaccination, due to the limited vaccination rate (the vaccination rate in Taiwan as of 5 June 2021 was 2.85%), such vaccination measures did not effectively contain the diffusion of B.1.1.7. A series of stricter measures were taken to control the B.1.1.7 spread, such as tightening the entry and exit measures, strengthening inspections, and raising the epidemic alert level. Among these measures, the most effective control measure was the raising of the epidemic alert level to the Level 3 epidemic alert for all of Taiwan's regions. Within the following 1–2 weeks (19–26 May 2021 and 26 May to 2 June 2021) after the implementation of the Level 3 epidemic alert for all of Taiwan's regions, the overall onset risk in Taiwan was reduced only by 0.54% and 7.10%, respectively (Figure 5a and Table 3). Furthermore, the *q*‐statistic results showed that human mobility played an important role in the spread of B.1.1.7 in 368 districts with a *q* value of 0.97 (P: 0.008) (Table 4). Therefore, although overall daily human mobility in Taiwan had gradually dropped below 50% (Figure 5b), the daily human mobility in some cities such as New Taipei at the center of the epidemic still maintained 50%–90% of the base human mobility (Figure 5c). Hence, the controlling effect of this measure appears to be limited.

**Table 3:** The Reduction Percentage of Overall Onset Risk in 368 Districts After the Raising of the Epidemic Alert Level to the Level 3 Epidemic Alert for all of Taiwan's Regions From 19 May to 2 June 2021

	19 May 2021	26 May 2021	2 June 2021
The relaxation of the quarantine and anti‐epidemic measures for flight crews of those airlines arriving in Taiwan	0.747	0.743 (reduction in overall onset risk: 0.54%)	0.694 (reduction in overall onset risk: 7.10%)

**Table 4:** The Determinant Power of the Human Mobility on the Risk of COVID‐19 Symptom Onset in 368 Districts of Taiwan

	Human mobility
*q*‐statistic	0.97
*p*‐value	0.008483

### 3.3 How to Control the Spatiotemporal Spread of B.1.1.7 Effectively by Using Epidemic Alert Measures

The Level 3 epidemic alert implemented in the Greater Taipei area and the entirety of Taiwan (Taiwan Centers for Disease Control, 2021e) seems to have played a limited role in controlling the spread of B.1.1.7 in 368 districts of Taiwan. Thus, one view (The Guardian, 2021) was proposed that the epidemic alert in all 368 districts should be raised to Level 4 epidemic alert to contain the epidemic more quickly and more effectively. Another opposite view (Adrian Kennedy, 2021) was that in cities other than the high‐onset‐risk Greater Taipei area, the Level 3 epidemic alert should be reduced to the Level 2 epidemic alert to partially restore normal economic and social activities in these cities. Therefore, based on the predicted risk of COVID‐19 symptom onset in the 368 districts of Taiwan, the impact of the different epidemic alert levels on the spread of B.1.1.7 was further analyzed. The impact was analyzed and compared based on three scenarios: (a) existing scenario under the Level 3 epidemic alert implemented from 15 May 2021 in the Greater Taipei area and throughout the whole of Taiwan; (b) the new scenario of the Level 4 epidemic alert for Taipei and New Taipei together with the Level 2 epidemic alert for the remaining 19 cities simulated and to be implemented on 15 May 2021 (i.e., lockdown for the districts of Taipei and New Taipei and general crowd control for the remaining 19 cities); and (c) the new scenario of the Level 2 epidemic alert for the whole of Taipei maintained after 15 May 2021. The latter two new scenarios were to be simulated based on different human mobility and social distancing levels.

**Figure 6:**
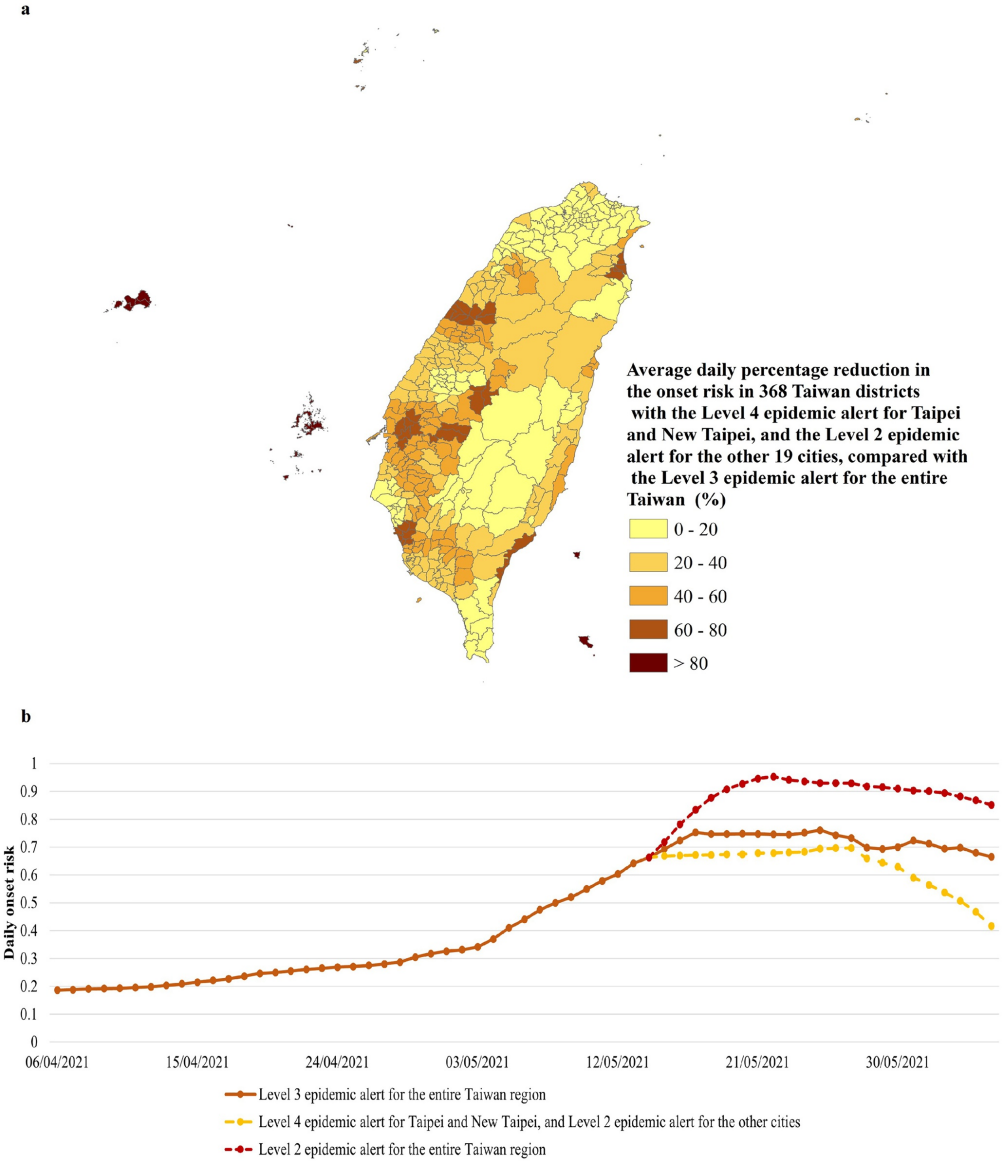
The risk of COVID‐19 symptom onset in the three epidemic alert scenarios (i.e., the current Level 3 epidemic alert for the entire Taiwan region, the Level 4 epidemic alert for Taipei and New Taipei together with the Level 2 epidemic alert for the remaining 19 cities, and the Level 2 epidemic alert for the entire Taiwan region) from 5 April to 5 June 2021. (a) The average daily percentage reduction in the onset risk in 368 Taiwan districts regarding the scenario of the Level 4 epidemic alert for Taipei and New Taipei together with the Level 2 epidemic alert for the remaining 19 cities, when compared with the scenario of the current Level 3 epidemic alert for the entire Taiwan region. (b) The overall onset risk in the three epidemic alert scenarios. The plotted values were computed from the predicted risk of COVID‐19 symptom onset in the three epidemic alert scenarios.

Compared with the risk of COVID‐19 symptom onset in the current scenario of Level 3 epidemic alert, in the new scenario of the Level 4 epidemic alert for Taipei and New Taipei together with the Level 2 epidemic alert for the remaining 19 cities on the same date, the onset risk in all 368 districts was obviously lower (Figure 6a). The reduction percentage in the onset risk was larger than 20%, 40%, 60%, and 80% in 119, 79, 35, and 14 districts, respectively (Figure 6a). This was achieved in up to 91.36% of all the districts (Figure 6a). Shown in this new scenario was the fact that the spread of B.1.1.7 had been more effectively contained. Moreover, the *q*‐statistic results (Table 5) showed that the epidemic alert level was an important influence on the spatial heterogeneity of onset risk in 368 districts with a *q* value of 0.23 (P: 0.000). By lockdown in these districts located in the Greater Taipei area from the source of B.1.1.7, it was effective to control the spread of B.1.1.7 from important nodes of mobility networks to the surrounding districts. The onset risk values in these surrounding districts were mostly reduced by more than 60% (Figure 6a). Under this circumstance, although other areas still maintained the basic flow control of Level 2 epidemic alert rather than upgrading to the closure of all places of business and public venues under the Level 3 epidemic alert, the promotion of the spread of B.1.1.7 was prevented.

**Table 5:** The Determinant Power of the Epidemic Alert Level on the Risk of COVID‐19 Symptom Onset in 368 Districts of Taiwan

	The epidemic alert level
*q*‐statistic	0.23
*p*‐value	0.000

The temporal variations of daily overall onset risk in Taiwan in the above three scenarios of the epidemic alert level were further used to analyze the effect of the different alert levels. Compared with the current scenario of the Level 3 epidemic alert, in the new scenario of the Level 4 epidemic alert for Taipei and New Taipei together with the Level 2 epidemic alert for the remaining 19 cities, the effects of controlling the spread of B.1.1.7 had mainly in all the following three aspects: a constant lower daily overall onset risk after the Level 4 alert, the delayed arrival of peaks of daily overall onset risk by 2 days (Figure 6b), and subsequent lower peak risks. Approximately 22 days after the Level 4 epidemic alert for Taipei and New Taipei together with the Level 2 epidemic alert for the remaining 19 cities were implemented, the daily overall onset risk decreased by 3.74%–59.65% (Figure 6b) compared with that under the current Level 3 epidemic alert. Similarly, a daily comparison of the predicted overall onset risk in Taiwan, under the Level 3 epidemic alert and the Level 2 epidemic alert, was also observed. It can be seen that a constant higher daily onset risk would have occurred if the Level 2 epidemic alert for all Taiwan regions was continued after 15 May 2021. The advanced arrival of peaks of the daily overall onset risk would have occurred earlier by 3 days, thus subsequently bringing the risks of higher peaks. In addition, the overall risk of COVID‐19 symptom onset had actually increased by 3.48%–24.24% compared with that under the current Level 3 epidemic alert (Figure 6b).

### 3.4 How to Effectively Control the Spatiotemporal Spread of B.1.1.7 by Means of the Oxford/AstraZeneca Vaccination

As of 5 June 2021, the Oxford/AstraZeneca vaccination rate range in Taiwan's 22 cities was still only 1.5%–9.48% (The Center for High‐performance Computing, 2021). The impact of the low vaccination rates in controlling the spatiotemporal spread of the onset risk appeared to be limited. Therefore, in the above three scenarios of the epidemic alert levels (Figure 6b), the effects of different Oxford/AstraZeneca vaccination levels in the spread of B.1.1.7 were simulated by the district‐level WKDE model in (a) the current vaccination rates in the 22 cities, (b) 5 times the current vaccination rates in the 22 cities [7.5%–47.4%], and (c) 10 times the current vaccination rates in the 22 cities [15%–94.8%]. Thus, the daily vaccination rate in each district at the different epidemic alert levels would be simulated by maintaining current rates and would increase by 5 and 10 times.

**Figure 7:**
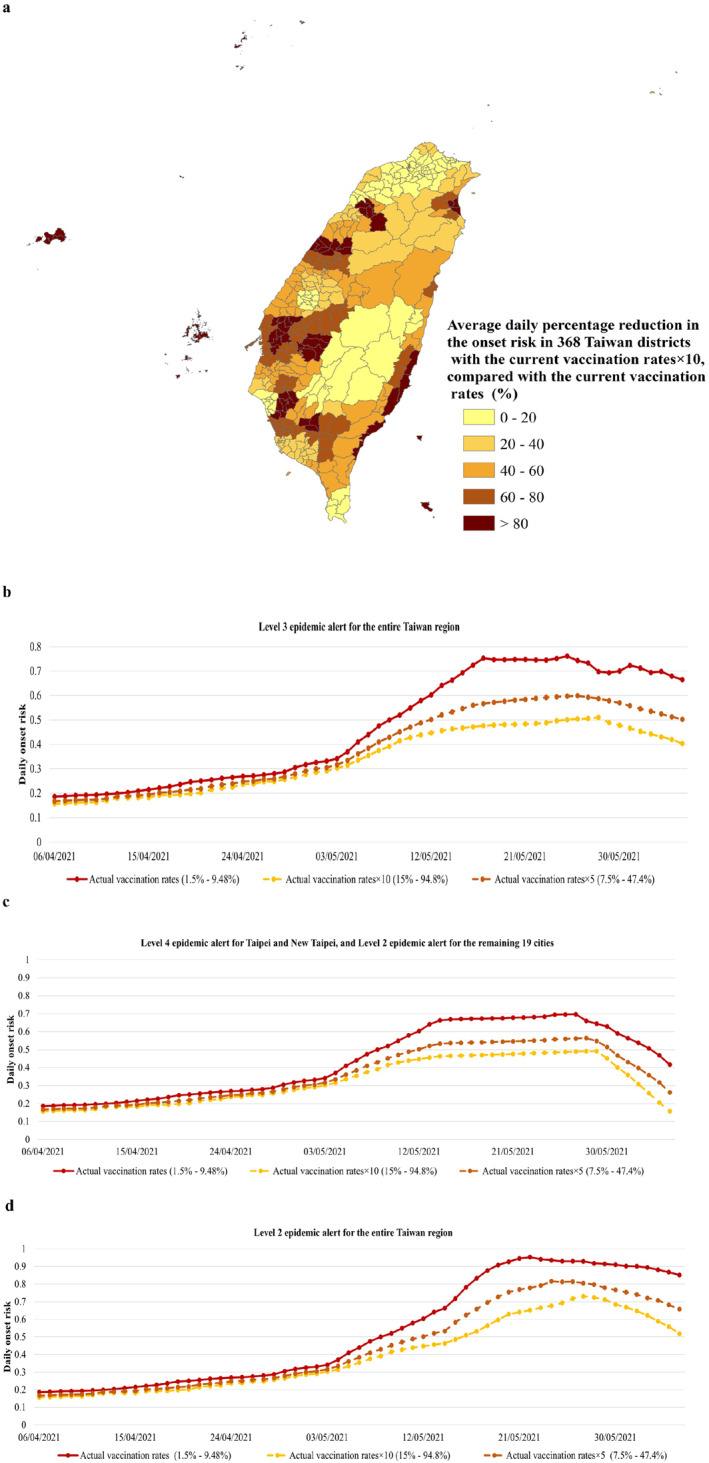
The risk of COVID‐19 symptom onset under the three scenarios (i.e., the current vaccination rates in 22 cities, 5 times the vaccination rates, 10 times the vaccination rate at the current Level 3 epidemic alert for the entire Taiwan region, the Level 4 epidemic alert for Taipei and New Taipei together with the Level 2 epidemic alert for the remaining 19 cities, and Level 2 epidemic alert for the entire Taiwan region) from 5 April to 5 June 2021. (a) The average daily percentage reduction in the onset risk in 368 Taiwan districts with 10 times the current vaccination rates in the scenario of the Level 4 epidemic alert for Taipei and New Taipei together with the Level 2 epidemic alert for the remaining 19 cities, compared with the current vaccination rates in the scenario of the Level 4 alert for Taipei and New Taipei together with the Level 2 epidemic alert for the remaining 19 cities. (b) The overall onset risk with the current vaccination rates, 5 times the vaccination rates, and 10 times the vaccination rates in the scenario of the current Level 3 epidemic alert for the entire Taiwan region. The plotted values were computed from the predicted risk of COVID‐19 symptom onset in 368 districts of Taiwan. (c) The overall onset risk with the current vaccination rates, 5 times the vaccination rates, and 10 times the vaccination rates in the scenarios of the Level 4 epidemic alert for Taipei and New Taipei together with the Level 2 epidemic alert for the remaining 19 cities. The plotted values were computed from the predicted risk of COVID‐19 symptom onset in the 368 districts of Taiwan. (d) The overall onset risk under the current vaccination rates, 5 times the vaccination rates, and 10 times the vaccination rates in the scenario of the Level 2 epidemic alert for the entire Taiwan region resulting from the district‐level weighted kernel density estimation model. The plotted values were computed from the predicted risk of COVID‐19 symptom onset in 368 districts of Taiwan.

In three scenarios of (a) the Level 3 epidemic alert throughout Taiwan, (b) the Level 4 epidemic alert for Taipei and New Taipei together with the Level 2 epidemic alert for the remaining 19 cities, and (c) the Level 2 epidemic alert for the whole of Taipei, the onset risk in all the 368 districts, which had 5–10 times the current vaccination rates [15%–94.8%], on the same date, was obviously lower than that with the current vaccination rate. For example, in the scenario of the Level 4 epidemic alert for Taipei and New Taipei together with the Level 2 epidemic alert for the remaining 19 cities, when compared with the Level 3 epidemic alert, a decrease in the onset risk further achieved up to 97.59% throughout all the 368 districts when the current vaccination rates were improved 10 times (Figure 7a). The reduction of the onset risk in 64 districts was even larger than 80% (Figure 7a). Moreover, the q‐statistic result (Table 6) also showed that the vaccine rate was an important influence on the spatial heterogeneity of onset risk in 368 districts with a *q* value of 0.38 (P: 0.000). The Level 4 epidemic alert of the Greater Taipei area brought the B.1.1.7 spread under control in the surrounding areas of important cities of the mobility network (Figure 7a). On this basis, an increase by 10 times, in the current vaccination rates in all the 368 districts, would further control the spread of B.1.1.7 in the surrounding districts of the main metropolitan areas (Figure 7a). The onset risk values in these areas appear to have been reduced by more than 60%.

**Table 6:** The Determinant Power of the Vaccine Rate on the Risk of COVID‐19 Symptom Onset in 368 Districts of Taiwan

	The vaccine rate
*q*‐statistic	0.38
*p*‐value	0.000

In the above three epidemic alert scenarios, the temporal variation of the daily overall onset risk values in Taiwan under the current vaccination rates of [1.5%–9.48%], 5 times the current vaccination rates of [7.5%–47.4%], and 10 times the current vaccination rates of [15%–94.8%] was further explored to reflect the effect of the improved vaccination rates (Figures 7b–7d): (a) A constant lower daily overall onset risk was found from 6.07% to 62.22% after a 5 or 10 times increase in the current vaccination rates. Due to the accumulation of differences in daily vaccination rates, this decreasing trend became more obvious over time; (b) the delayed arrival of peaks of the daily overall onset risk of 1–4 days with vaccination rates increased by 5 or 10 times (Figures 7b and 7c); (c) the subsequent lower peak risks from 14.40% to 32.97%. For example, at the Level 3 epidemic alert for the entire Taiwan region, if the cumulative vaccination rates from 5 April 2021 were increased by 10 times [15%–94.8%], the daily overall onset risk would decrease by 9.34%–39.41% (Figure 7b). The decreasing values of the daily overall onset risk were also increased from 6.07% to 37.26% with 5 times the current vaccination rates realized at [7.5%–47.4%]. However, in the scenario of the Level 4 epidemic alert for Taipei and New Taipei together with the Level 2 epidemic alert for the remaining 19 cities, the decreasing trend in the daily overall onset risk was more obvious (Figure 7c). Specifically, by 5 June 2021, with 5 times the current vaccination rates at [7.5%–47.4%] and 10 times the current vaccination rates of [15%–94.8%], the overall onset risk level dropped to a low‐medium risk level. Even if, in the scenario of the Level 2 epidemic alert for the whole of Taiwan (Figure 7d), with 5 times the current vaccination rates at [7.5%–47.4%] and 10 times the current vaccination rates of [15%–94.8%], the onset risk would also be reduced to the medium‐high risk or the medium risk level.

## 4 Conclusions

For the reopening world, an investigation into the emergence of the novel lineages of SARS‐CoV‐2 and the tracking of their spatial spread dynamics are not only necessary, but also essential regarding the enablement of various regions to take appropriate epidemic prevention measures and thereby effectively respond to new variants. Hence, this study provides an analysis of the spatiotemporal dynamics of the B.1.1.7 spread at the within‐city scale, which can explain the mechanism of spatial spread of B.1.1.7 in an increased in‐depth manner. Of importance, in this respect, is that this study uses the proposed district‐level WKDE model to enable the prediction of the risk of COVID‐19 symptom onset, in order to retrospectively analyze the entire dynamic process of the spatiotemporal spread of B.1.1.7 during the reopening process. The effect of relaxation and tightening of the prevention measures on the emergence and spread of B.1.1.7 is then investigated. Furthermore, how to control the spatiotemporal spread of B.1.1.7 by integrating epidemic prevention measures and vaccination levels is also analyzed. Specifically, the spatiotemporal dynamics of the B.1.1.7 spread in Taiwan is explored.

Based on daily human mobility, vaccination rates, and social distancing factors at the within‐city scale to strengthen the district‐level WKDE model, it has been seen that the impact of spatiotemporal differences of symptom onset risk, interregional mobility, NPI measures, and vaccination to the new SARS‐CoV‐2 lineage growth can be quickly assessed. The results are shown in this study: (a) For all the 368 districts in Taiwan with high human mobility and low vaccine rates before the emergence of B.1.1.7, even the partial relaxation of the entry policy for specific imported groups, during the process of reopening, could lead to the emergence and rapid spatiotemporal spread of B.1.1.7. The high onset risk districts expanded by 1.7 times with a continuous increase by 21.4%–104.65% as regards the overall onset risk level over the next 1–3 weeks. (b) After the emergence of B.1.1.7 and because of the close communication between high‐onset‐risk areas and other cities, high human mobility was not interrupted, and B.1.1.7 spread along the center of the mobility network to the important cities of the mobility network in Taiwan. As a result, seven metropolitan areas accounting for more than 74% of Taiwan's population became the high‐onset‐risk areas severely affected by B.1.1.7. However, B.1.1.7 appeared to have a limited impact in rural areas far from the main mobility network. (c) B.1.1.7 had significant spatiotemporal heterogeneity and human mobility played an important role in the transmission of B.1.1.7. This factor also supports the findings of some previous studies based on the relationship between mobility and COVID‐19 transmission (Chen et al., 2021; Jia et al., 2020; Levin et al., 2021; Li et al., 2021; Nouvellet et al., 2021; Sachak‐Patwa et al., 2021). The above conclusion can act as a guide to any region considering reopening to formulate precise entry measures and hence prevent the emergence of new variants in that particular region and thereby cutting possible networks of spread.

Through the simulation and comparison of how to control the spread of B.1.1.7 under different scenarios with different levels of epidemic prevention measures and vaccination rates, this current study also provides a scientific reference for areas affected by SARS‐CoV‐2 variants. The results regarding recorded attempts to control the spatiotemporal spread of B.1.1.7 in the 368 districts of Taiwan show that:

1) The epidemic alert measures were proved to have a significant association with B.1.1.7 transmission. Compared with the implementation of current alert measures such as the closing of all places of business and public venues throughout Taiwan, imposing lockdown in the high‐onset‐risk districts where the source of B.1.1.7 is located could possibly more effectively control the spatiotemporal spread of B.1.1.7 as in this way. The onset risk is more likely to be reduced by up to 91.36% throughout all districts and in particular, the surrounding districts of important cities within mobility networks. Further, the onset risk values in most of such surrounding districts could be reduced by more than 60%. In other districts, at corresponding times, in general crowds and gatherings, control measures could be taken to reduce the impact on further normal socioeconomic activities and public services. Such measures would control further aggravation of the spatial spread of B.1.1.7.
2) On the basis of the above effective epidemic alert measures, the Oxford/AstraZeneca vaccination rate in each district was raised to a certain higher level from the current vaccination rates to 5 and 10 times. The vaccination rate was found to be strongly associated with the spatiotemporal variations of B.1.1.7. Thus, giving further promise that the spatiotemporal spread of B.1.1.7 could be controlled more effectively by further reducing the onset risk by up to 97.59% throughout all districts, particularly the medium‐onset‐risk districts outside the metropolitan areas. In addition, the peak of the overall onset risk could also be reduced from 14.40% to 32.97%, hence delaying the arrival from 1 to 4 days. A retrospective cohort study in London has proved that the Oxford/AstraZeneca vaccine was effective at reducing the risk of testing positive for COVID‐19 in an urban UK population. The findings of this study support the finding of this cohort study (Glampson et al., 2021). Hence, it can be assumed that the key to control the spatial spread of SARS‐CoV‐2 variants is implementing lockdowns at all high‐risk areas as soon as possible and at the same time increasing the vaccination rates as much as possible throughout the whole area. For areas other than high‐risk areas, general epidemic prevention measures such as gathering and crowd control measures are appropriate to maintain basic local socioeconomic activities.

Various regions have attempted to reopen in recent months, and these regions have eased their COVID restrictions and social distancing rules. Unfortunately, the reopening was not without its challenges. These regions that have attempted to reopen are facing a renewed COVID‐19 outbreak caused by the new variants, and therefore have to resume the applicable COVID rules shortly. Nowadays, in the context of the global spread of the new SARS‐CoV‐2 lineage B.1.617 (Delta), some countries still have continued their reopening plans by removing all legal restrictions on social contact. As the study provides a comprehensive investigation about the spatiotemporal dynamics of SARS‐CoV‐2 variant B.1.1.7, we hope that this study can assist countries and regions to reopen strategically. Moreover, for countries and regions where the new SARS‐CoV‐2 variants have appeared, the scientific findings of this study can also assist to control the spatiotemporal spread of new SARS‐CoV‐2 variants by integrating the prevention measures and vaccination.

## Conflict of Interest

The authors declare no conflicts of interest relevant to this study.

## Data Availability

Data on COVID‐19 cases can be accessed at https://sites.google.com/cdc.gov.tw/2019ncov/taiwan. Apple Mobility data can be accessed at https://covid19.apple.com/mobility. Google Mobility data can be accessed at https://www.google.com/covid19/mobility/. The vaccination rates data in 22 cities of Taiwan can be accessed at https://www.cdc.gov.tw/Category/List/P2pYv_BSNAzqDSK8Qhllew. Source data for all figures and tables in the manuscript can be accessed at https://doi.org/10.5281/zenodo.5614418 (Tong et al., 2021).
